# Catheterization in a patient with end-stage renal disease through persistent left superior vena cava: a rare case report and literature review

**DOI:** 10.1186/s12882-019-1339-5

**Published:** 2019-06-04

**Authors:** Huisi He, Bingyang Li, Yiyi Ma, Yuqiang Zhang, Chaoyang Ye, Changlin Mei, Shengqiang Yu, Bing Dai, Yawei Liu

**Affiliations:** 1 Naval Clinical Medicine Grade 2014, Basic Medical College, Naval Medical University, Shanghai, 200433 People’s Republic of China; 2 Department of Nephrology, Changzheng Hospital, Naval Medical University, Shanghai, 200433 People’s Republic of China

**Keywords:** Persistent left superior vena cava, Hemodialysis catheter, Case report

## Abstract

**Background:**

Persistent left superior vena cava (PLSVC) is a common vena cava malformation, and drains blood into the right atrium via the dilated coronary sinus in most cases. It is usually asymptomatic and detected incidentally during invasive procedures or imaging. Whether the hemodialysis catheters can be placed in PLSVC is still controversial now (Stylianou et al. Hemodial Int 11:42-45, 2007).

**Case presentation:**

Here we report a rare case of catheterization through PLSVC in an end-stage renal disease (ESRD) male patient whose PLSVC connected with pulmonary vein with insufficient blood flow eventually. Among the other 28 cases included in the literature review, 16 cases were non-tunneled catheter and 12 cases were cuffed, tunneled catheter and most of them could provide adequate blood flow.

**Conclusion:**

PLSVC is a rare malformation and mostly asymptotic, we believe that PLSVC drains blood into the right atrium with enough inner diameter and blood flow can serve as an alternative site for conventional dialysis access. However, the feasibility of hemodialysis catheterization through it and measures to avoid serious complications are still needed to be discussed.

## Background

Persistent left superior vena cava (PLSVC), known as the residual left superior vena cava, is the most common type of vena cava malformations despite its low incidence. In most cases, PLSVC is clinically asymptomatic due to the lack of hemodynamic abnormalities and is almost always found in invasive procedures or imaging.

Reliable and high-quality vascular access which can provide adequate extracorporeal blood flow is a prerequisite for hemodialysis and serves as a crucial factor for prognosis. Non-cuffed and cuffed, tunneled central venous hemodialysis catheter are both preferred choices for end-stage renal disease (ESRD) patients who have an urgent need for hemodialysis, especially when arteriovenous fistula or graft are both unavailable.

The presence of PLSVC brings difficulties and risks for central venous catheterization. Whether the hemodialysis catheters can be placed in PLSVC is controversial until now. Here we report a rare case of hemodialysis catheterization in a patient with ESRD through PLSVC, but it ended with insufficient blood flow compared to the previous case reports.

## Case presentations

A 54-year-old hemodialysis patient with a history of multiple central venous catheterizations, arteriovenous fistula, and graft operations was admitted to our unit for the creation of permanent vascular access. After initial screening, an arteriovenous fistula (AVF)/arteriovenous graft (AVG) was deemed not possible due to exhausted vasculature of both arms, and a cuffed, tunneled hemodialysis catheter was optioned to be chosen. The right internal jugular vein (IJV) catheterization was attempted under sterile conditions, but the guide-wire could not be advanced more than 10 cm, and the right IJV catheterization was abandoned due to consideration of potential critical stenosis. The left IJV was catheterized with a cuffed, tunneled hemodialysis catheter (14.5F, 36 cm, Palindrome) thereafter without any complication.

**Fig. 1:**
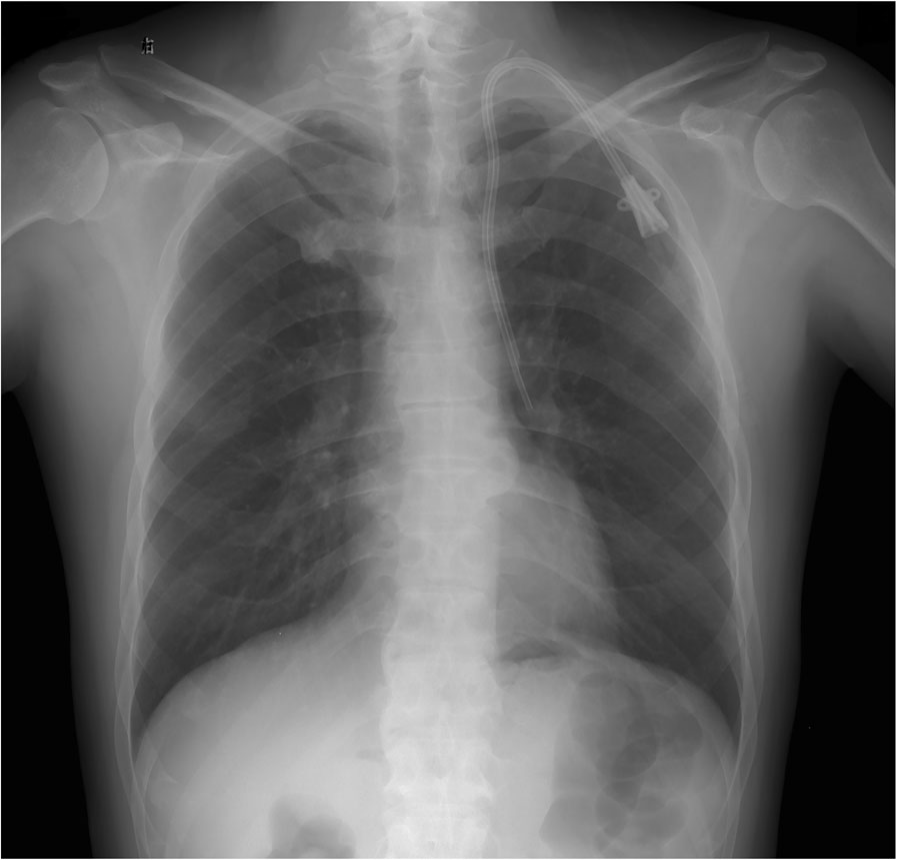
Postoperative chest radiograph showed the location of the cuffed, tunneled hemodialysis catheter and its abnormal path

**Fig. 2:**
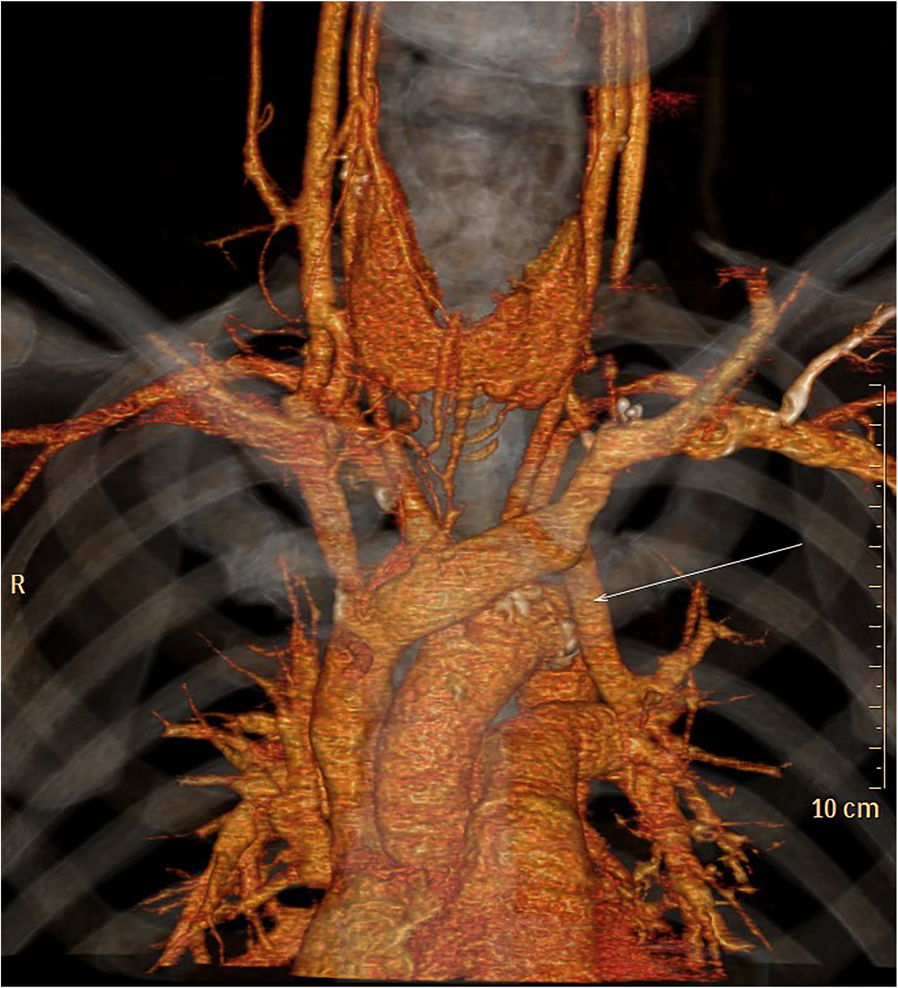
CTA of central vein and three-dimensional reconstruction confirmed PLSVC which connected with pulmonary vein

Postoperative chest radiograph showed that the catheter was descending straight through the left border of the mediastinum (Fig. 1). Further computed tomography angiography (CTA) of central veins after removal of the hemodialysis catheter, with three-dimensional reconstruction of vessels, revealed the initial segment of the left IJV was stenosed and an abnormal vessel on the left of the aorta drained blood into the left atrium via pulmonary vein. The vascular malformation of PLSVC was confirmed (Fig. 2).

**Fig. 3:**
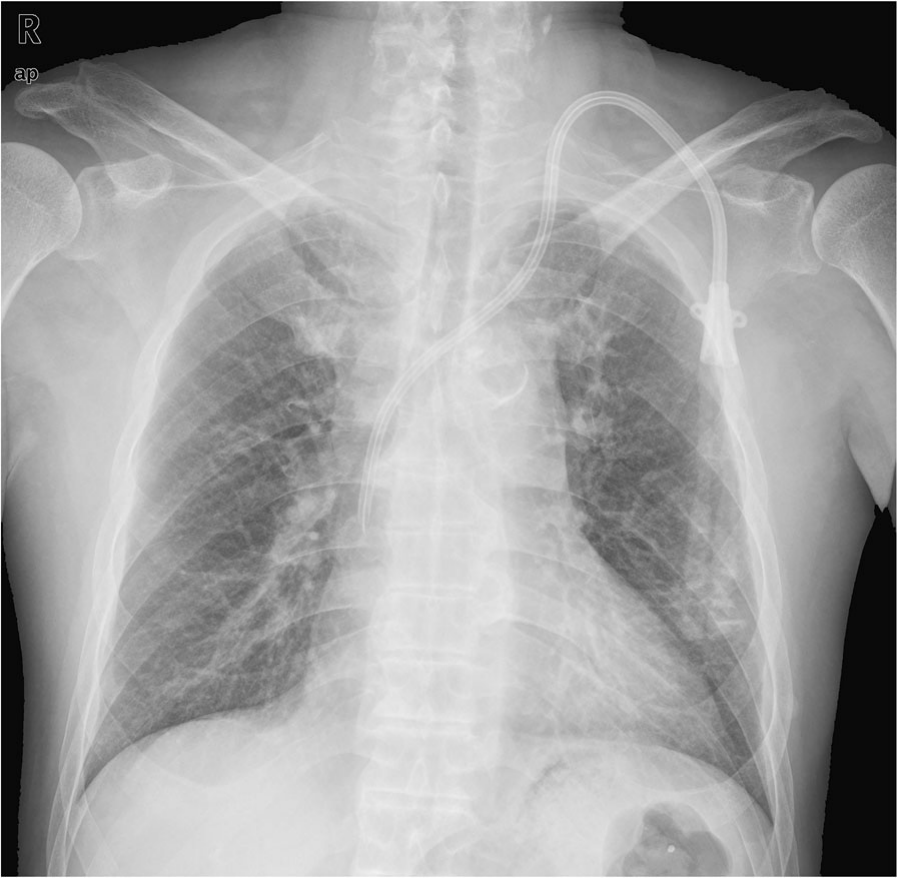
The chest radiograph of the cuffed, tunneled hemodialysis catheter used now

Finally, we replaced a cuffed, tunneled catheter through the right IJV after DSA-guided balloon dilatation of right brachiocephalic venous stenosis. It was removed due to decreasing blood flow and catheter-related bloodstream infection 3 years later. Thereafter, a new cuffed, tunneled catheter was placed in the left IJV which went through right superior vena cava into the right atrium under digital subtraction angiography (DSA) (Fig. 3). Until now, this patient has conducted hemodialysis through the catheter with blood flow around 300 mL/min for 4 years.

## Discussion and conclusion

PLSVC is the most common kind of congenital malformations in the thoracic vessels. It was first reported by Edwards et al. [1] in 1950 and the latest studies show that the incidence of this deformity is about 0.1–0.5% of the total population, [2] of which about 10% of patients with congenital heart abnormalities [3, 4].

**Fig. 4:**
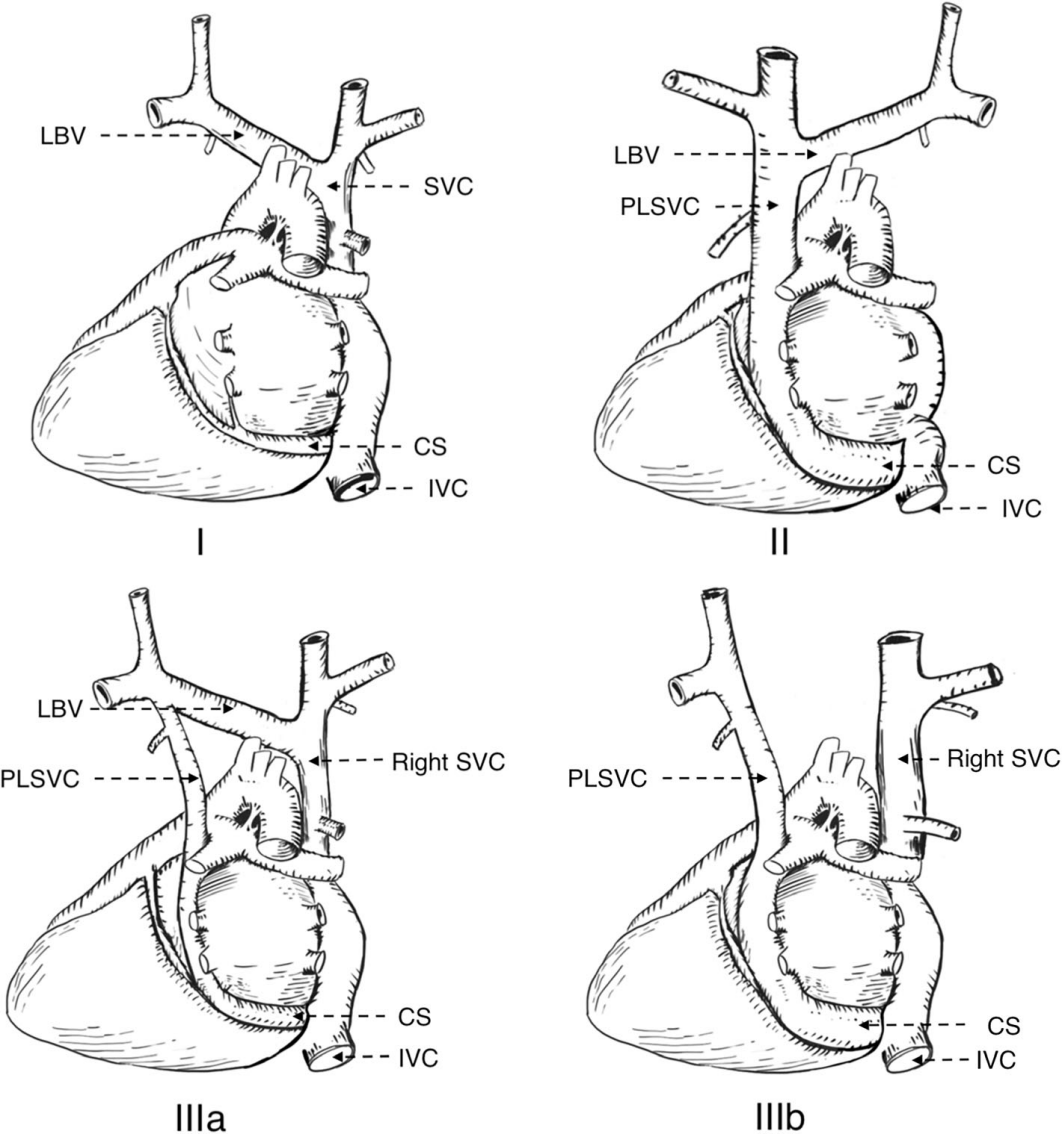
Schummer’s classification of superior vena cava in dorsal view (SVC: Superior vena cava PLSVC: Persistent left superior vena cava LBV: Left brachiocephalic vein CS: Coronary sinus IVC: Inferior vena cava)

Human left superior vena cava originates in the third week of the embryonic period, and then the left anterior cardinal vena cava gradually atrophies with embryonic development and finally degenerates into the ligament of Marshall. If the degeneration is not complete, then the remains of a pipeline structure after birth is PLSVC. Some clinicians advocate that it associates with chromosomal aberration, congenital cardiac defect, and extracardiac anomalies might be detected at follow-up [5]. Schummer [6] raised the most recognized classification of the supracardial venous system according to anatomic relationships of superior vena cava and its adjacent (Table 1, Fig. 4). The patient in our case had a type IIIa venous malformation.

**Table 1:** Schummer’s classification of superior vena cava

Types	Characteristics
I	Normal superior vena cava anatomy
II	Only PLSVC exists, without the right superior vena cava
IIIa	PLSVC and the right superior vena cava exist, with left brachiocephalic vein between both sides
IIIb	PLSVC and the right side of the superior vena cava, withoutleft brachiocephalic vein between both sides

**Fig. 5:**
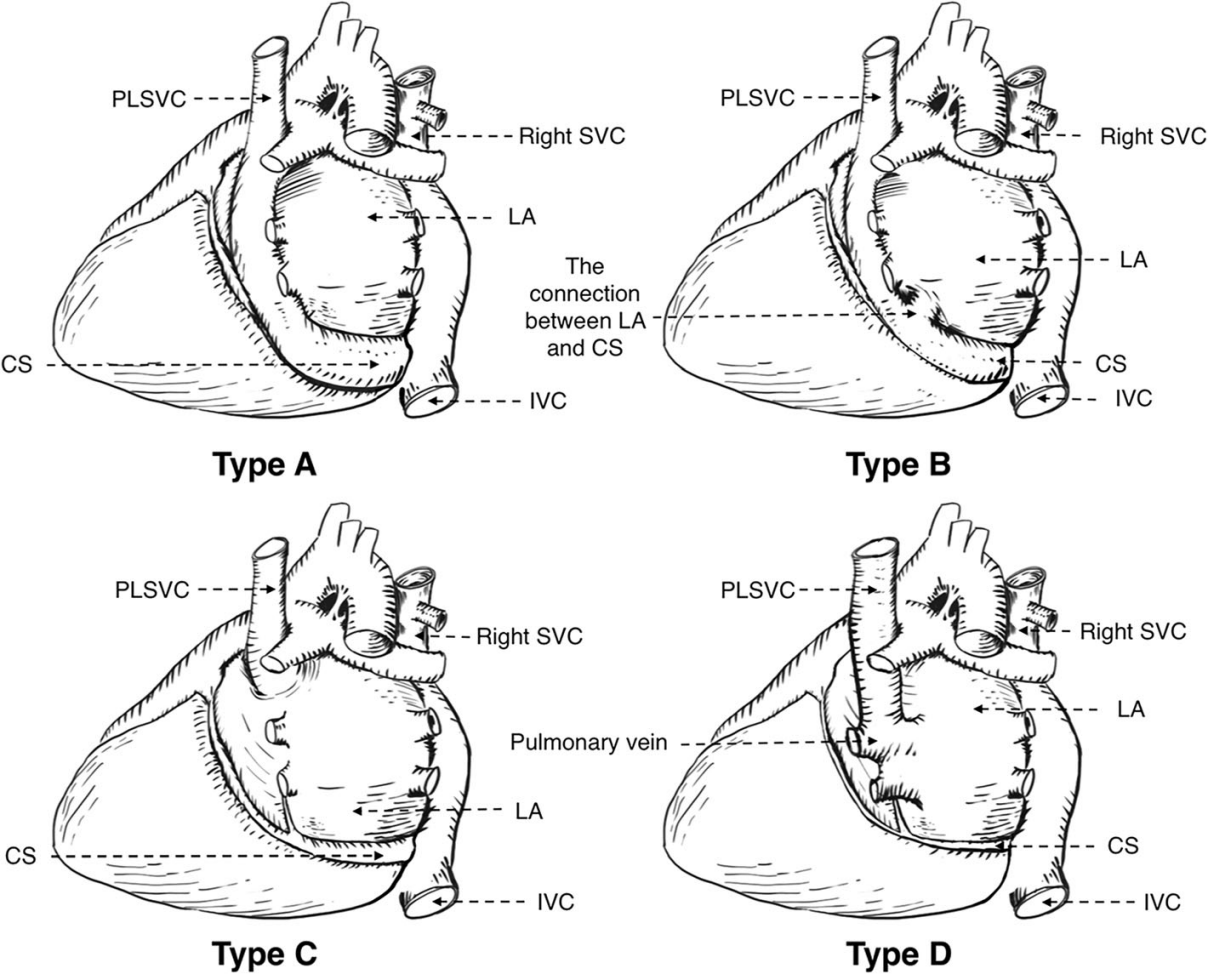
Zhu’s classification of PLSVC in dorsal view (SVC: Superior vena cava PLSVC: Persistent left superior vena cava CS: Coronary sinus LA: Left atrium IVC: Inferior vena cava)

Ninety-two precent % of PLSVC patients drain blood into the right atrium via the dilated coronary sinus, [7] most of them are asymptomatic and have no hemodynamic abnormalities. In most cases, it’s hard to be detected by physical examination and it is always noticed accidentally during imaging or the process of intravascular invasive procedure such as pacemaker implantation, PICC, cardiac electrophysiological examination and central venous hemodialysis catheterization. However, some patients still show abnormal sinus rhythm or bradycardia at the very beginning. In these cases, the patients might undergo pacemaker implantation because of sick sinus syndrome resulting from histological abnormalities caused by an enlarged coronary sinus [8, 9]. Another 8% of patients drain blood into left atrium may have obvious clinical cyanosis due to the left to right shunt, and those people always suffer from septal defect, ventricular septal defect or other cardiovascular malformations [10, 11]. This patient’s PLSVC drains blood into the left atrium via pulmonary vein (Type D in Zhu’s classification of PLSVC), but he doesn’t have congenital heart disease and cyanosis which may result from low shunt flow volume (Table 2, Fig. 5) [12].

**Table 2:** Zhu’s classification of PLSVC

Types	Characteristics
A	PLSVC drains blood to right atrium via coronary sinus
B	PLSVC drains blood to right atrium via coronary sinus with partial right-to-left shunt
C	PLSVC drains blood to left atrium directly with right-to-left shunt
D	PLSVC is directly connected to left pulmonary vein (coronary sinus absent)

Can persistent left superior vena cava be used in the hemodialysis catheterization? After a careful literature review, totally 28 cases with hemodialysis catheterization through PLSVC were reported. The details of case reports with hemodialysis catheterization through PLSVC are shown in Table 3. Among them, 16 cases were non-tunneled catheter and 12 cases were cuffed, tunneled catheter. Most of them were type III PLSVC with indwelling catheters in left IJV. The previous history of pacemaker implantation was also notable in the latest case we reported [39]. Among these cases, most operations were completed safely, and hemodialysis catheters met the needs of hemodialysis during the maximum 32-month dwelling time. There was one case reported severe hypotension, bradycardia, and cardiac-respiratory arrest after three times successful hemodialysis. Although the correlation between catheterization and arrhythmia was uncertain, the catheter was removed after the fourth hemodialysis was performed [17]. In another case, rare complication pericardial effusion and bilateral pleural effusions were confirmed by chest computed tomogram since short of breath developed 24 h after catheterization and hemodialysis. This catheter was removed by the cardiothoracic surgeon for safety [28]. In a recently released case, stagnation of blood flow and thrombus formation was found due to a large catheter caliber-to-vein ratio, which resulted in catheter removal after 4 h [38]. Our case is the first hemodialysis patient with PLSVC that drains blood into the left atrium via pulmonary vein, which leads to insufficient blood flow after catheterization. From this rare case and previously reported cases, we raise some concerns about catheterization in PLSVC.

**Table 3:** The details of case reports with hemodialysis catheterization through PLSVC

Authors & Year	Study type	Patient’s Gender & Age	The reason of catheterization	The type of SVC	The type of catheter	Catheter Function & Blood Flow (ml/min)	The duration of catheterization	The outcome of catheter or patient	Intraoperative & postoperative complications	Additional anatomical variations
Kim et al., 1999 [13]	Letter to editor	28, male	ESRD	Type IIIb	Non-tunneled (left SCV)	Good, 200 mL/min	Unclear but carried out 3 times	Catheter was removed when AVF matured	Not observed	Not observed
Paulter et al., 1999 [14]	Case report	83, male	ESRD due to DM and HTN	Unclear	Non-tunneled (left IJV)	Good, Unclear	Unclear	Unclear	Not observed	Not observed
Radovic et al., 2002 [15]	Letter to editor	31, female	ESRD due to membranoproliferative glomerulonephritis	Type IIIa	Non-tunneled (left IJV)	Good, 220 mL/min	4 weeks	Catheter was removed when AVG was cannulated	Not observed	Not observed
De la Prada et al., 2002 [16]	Case Report	45, male	ESRD due to DM	Type III (a or b)	Cuffed, tunneled (right IJV)	Good, 250 ml/min	More than 3 months	Unclear	Not observed	Not observed
Dionison et al., 2003 [17]	Case report	61, female	ESRD due to DM	Type IIIb	Cuffed, tunneled (left IJV)	Good, Unclear	Unclear but carried out 4 times	Catheter was removed because of severe arrhythmia	Severe hypotension and bradycardia, cardiac-respiratory arrest	A solitary pelvic kidney
Kuppusamy et al., 2004 [18]	Case report	75, female	AKI due to ischemic tubular necrosis	Type IIIb	Non-tunneled (left IJV)	Good, Unclear	Unclear	Unclear	Not observed	Not observed
Stylianou et al., 2007 [19]	Case report	80, female	ESRD due to DM	Type III (a or b)	Non-tunneled (left IJV)	Good, Unclear	1 month	Catheter was removed when AVG was cannulated	Not observed	Anomalous pulmonary vein drainage
Orija et al., 2009 [20]	Case report	72, male	ESRD	Type III (a or b)	Cuffed, tunneled (right IJV)	Good, Unclear	Unclear	Unclear	Not observed	Not observed
Parreira et al., 2009 [21]	Case report	50, unclear	ESRD	Unclear	Cuffed, tunneled (left IJV)	Good, Unclear	Unclear	Unclear	Not observed	Not observed
Jang et al., 2009 [22]	Case report	68, male	ESRD	Unclear	Non-tunneled (left IJV)	Good, 230 mL/min	Unclear	Unclear	Not observed	Not observed
Lim et al., 2010 [23]	Case report	58, male	ESRD due to DM	Unclear	Cuffed, tunneled (left IJV)	Good, Unclear	5 months	Catheter was removed when AVF matured	Not observed	Aortic coarctation
Sriramnaveen et al., 2010 [24]	Letter to editor	50, male	ESRD due to HTN	Type IIIa	Non-tunneled (left IJV)	Good, Unclear	Unclear	Unclear	Not observed	Not observed
Messina et al., 2011 [25]	Case report	Unclear	ESRD with complete obstruction of central venous vessels	Type III (a or b)	Cuffed, tunneled (left IJV)	Good, Unclear	15 months	Catheter was replaced with a longer one at 12 months	Not observed	Not observed
Kute et al., 2011 [26]	Case report	45, female	ESRD due to DM and HTN	Type III (a or b)	Cuffed, tunneled (left IJV)	Good, 250 mL/min	2 months	Catheter was removed when AVF matured	Not observed	Not observed
Wong et al., 2013 [27]	Case report	Unclear, male	ESRD due to systemic lupus erythematosus	Type IIIa	Non-tunneled (left IJV)	Good, Unclear	3 months	Patient died of pancytopenia and infective endocarditis	Not observed	Not observed
Balasubramanian et al., 2014 [28]	Case report	57, male	AKI	Unclear	Non-tunneled (left IJV)	Good, Unclear	4 h	Catheter was removed by cardiothoracic surgeon	Breathlessness, bilateral pleural effusions, subcutaneous, emphysema, pericardial effusion	Not observed
Lui et al., 2014 [29]	Case report	61, male	ESRD due to DM	Unclear	Cuffed, tunneled (left IJV)	Good, Unclear	6 months	Catheter was removed when AVF matured	Not observed	Not observed
Kukavica et al., 2014 [30]	Letter to editor	71, male	ESRD	Unclear	Non-tunneled (left IJV)	Good, Unclear	4 months	Patient died of cerebrovascular stroke, cardio-respiratory insufficiency and cardiac arrest	The failed first two insertions and mild initial resistance during the third insertion	Not observed
Dubey et al., 2014 [31]	Letter to editor	35, male	ESRD (waiting for another renal transplantation)	Type II	Non-tunneled (right IJV)	Good, Unclear	Unclear	Unclear	Not observed	Not observed
Jaffer et al., 2015 [32]	Case report	58, female	AKI due to acute tubular necrosis	Type IIIa	Cuffed Tunneled (right IJV)	Good, Unclear	Unclear	Unclear	Not observed	Horseshoe kidney
Sahutoglu et al., 2016 [33]	Case reports	80, male	ESRD (acute peritonitis due to peritoneal dialysis)	Type II	Non-tunneled (left IJV)	Good, 300–350 mL/min	3 months	Catheter was removed when AVF matured	Not observed	Not observed
		35, male	ESRD due to DM and HTN	Type II	Non-tunneled (Right IJV)	Good, 300–350 mL/min	2 months	Catheter was removed when AVF matured	Not observed	Not observed
Zhou et al., 2016 [34]	Case report	63, female	ESRD	Unclear	Cuffed, tunneled (left IJV)	Good, Unclear	9 months	Unclear	Not observed	Not observed
Ricciardi et al., 2017 [35]	Case report	33, female	ESRD	Unclear	Cuffed, tunneled (left IJV)	Good, Unclear	32 months	Unclear	Not observed	Cleft lip and palate, uterus bicornis, congenital left hip dislocation and a left inferior vena cava
Boodhun et al., 2018 [36]	Case Report	28, male	ESRD	Type IIIb	Non-tunneled (left IJV)	Good, Unclear	Unclear	Catheter was removed when permanent left femoral catheter was placed	Not observed	Not observed
Anvesh et al., 2018 [37]	Case Report	35, male	ESRD due to HTN	Type IIIb	Non-tunneled (left IJV)	Good, Unclear	Unclear	Unclear	Not observed	Not observed
Kawasaki et al., 2018 [38]	Case report	66, female	ESRD due to DM and HTN	Unclear	Non-tunneled (left IJV)	Removed before use	4 h	Thrombus formation in the catheter lumen when removed	Not observed	Not observed
He et al., 2018 [39]	Case report	88, female	ESRD due to HTN	Type II	Cuffed, tunneled (right IJV)	Good, 220 mL/min	16 months	Patient died of gastrointestinal hemorrhage	Not observed	Not observed

*ESRD* end-stage renal disease, *HTN* hypertension, *DM* diabetes, *AVF* arteriovenous fistula, *SCV* subclavian vein, *IJV* internal jugular vein, *PLSVC* persistent left superior vena cava

Firstly, the operators should raise awareness of cardiovascular abnormalities during the central venous access. For suspected patients with positive symptoms and signs, echocardiography should perform as soon as possible. The direct signs are the existence of the duct-like structure and the blood flow spectrum in the left upper part of the chest, and the indirect sign is the dilated coronary sinus [40]. In addition, unexplained tricuspid atrial systolic murmur and right atrial enlargement should arouse attention. Localized bullae in front of the mediastinum in chest radiography is an important sign of early screening and echocardiography can be the primary screening method. Cardiac catheterization procedure is the gold standard for the diagnosis of PLSVC. However, its invasiveness, radioactivity prohibits clinical use. Thoracic enhanced CTA might serve as an alternative.

Secondly, left IJV is a preferred cannulation site for hemodialysis catheterization through PLSVC, especially for those patients with absent right superior vena cava. Traditionally, right IJV cannulation is generally preferred in hemodialysis patients due to its straight path directly into the superior vena cava and fewer complications compared with other positions. Nevertheless, in these PLSVC without right superior vena cava cases, since the right IJV and subclavian vein drains blood into PLSVC via the right brachiocephalic vein, traditional right IJV cannulation may encounter difficulties and acute complications normally met in left IJV cannulation. Central vein perforation, pneumothorax, and artery puncture all have been reported in previous cases, which mostly caused by force during the operation without the sense of cardiovascular malformations. So, whenever any resistance is met with forwarding the guidewire or the peel-away sheath, do not push by force, what you need is to pull it out and reassess vascular condition (especially for PLSVC with absent right superior vena cava). Detailed history survey, preoperative imaging screening, intraoperative fluoroscopic guidance, and postoperative chest radiograph assessment for suspected patients are priority points to avoid serious complications.

Thirdly, whether a hemodialysis catheter can be placed in PLSVC is still controversial until now [19]. Our case proved that the PLSVC which rarely drains blood into the left atrium via pulmonary vein or left-to-right shunt cannot be used to conduct hemodialysis because of obvious hemodynamic abnormalities and insufficient blood flow. In most cases, PLSVC flowed back into the right atrium through the coronary venous sinus. Although few complications were reported in the placement of a non-tunneled hemodialysis catheter through PLSVC (Table 3), hemodynamic changes after indwelling catheters in those patients potentially may lead to angina pectoris, arrhythmia, stroke, cardiac arrest due to coronary sinus irritation. In severe cases, it may threaten the patients’ life [19, 22, 41]. Some nephrologists believe that PLSVC is relatively thin and the blood flow is not enough to maintain long-term hemodialysis, and the locally generated turbulence may increase the probability of thrombosis and arrhythmia. However, if the diameter of PLSVC and blood flow were sufficient, with stably flowed back through the coronary venous sinus into the right atrium, it was feasible to dwell a hemodialysis catheter in PLSVC for long-term hemodialysis. We believe that after an accurate assessment of intrathoracic vessels including the inner diameter of PLSVC via preoperative imaging, a PLSVC can serve as an alternative site for conventional dialysis access.

However, the location of the catheter tip remains to be elucidated. The tip of the cuffed, tunneled hemodialysis catheter is normally positioned within the right atrium or at the junction of superior vena cava and right atrium. For PLSVC patients, the right atrium is inaccessible and the placement of catheter tip in the left superior vena cava that is close to the coronary sinus might cause arrhythmia, so we think that the lower left superior vena cava with adequate blood flow and negative cardiac effect might be an optimal choice.

PLSVC is a rare and asymptotic malformation, so the early detection and diagnosis before hemodialysis catheterization are quite difficult. Detailed history survey, echocardiography and preoperative imaging screening are the priority points to identify suspect patients. Rarely, the PLSVC which drains blood into the left atrium via pulmonary vein or left-to-right shunt should be excluded. During the surgery, intraoperative ultrasound and fluoroscopic guidance are strongly recommended if available. Performing catheterization carefully, position it properly and do not push it by force may help to avoid serious complications. We believe that PLSVC drains blood into the right atrium with enough inner diameter and blood flow can serve as an alternative site for conventional dialysis access. Besides, the preferable location of the catheter’s tip with minor hemodynamic effect remained to be determined.

## Abbreviations

AVG: arteriovenous graft
CTA: Computed tomography angiography
DSA: Digital subtracted angiography
ESRD: End-stage renal disease
IJV: Internal jugular vein
PLSVC: Persistent left superior vena cava
